# Special Issue “Nanotechnology to Overcome the World’s Most Critical Health Issues: Liposomes and Beyond—A Themed Issue Dedicated to Professor Yechezkel Barenholz”

**DOI:** 10.3390/molecules28124788

**Published:** 2023-06-15

**Authors:** Marina A. Dobrovolskaia, Kirill A. Afonin

**Affiliations:** 1 Nanotechnology Characterization Laboratory, Cancer Research Technology Program, Frederick National Laboratory for Cancer Research Sponsored by the National Cancer Institute, Frederick, MD 21701, USA; 2 Nanoscale Science Program, Department of Chemistry, University of North Carolina Charlotte, Charlotte, NC 28223, USA

This Special Issue is intended to celebrate Professor Yechezkel Barenholz’s distinguished achievements. Professor Barenholz is Professor Emeritus of the Hebrew University in Jerusalem, Israel. He joined the University’s faculty in 1968, received his PhD in 1971, and became a Professor in 1981. Throughout his career, Prof. Barenholz has taught young scientists at leading universities worldwide. One of the prominent achievements in Prof. Barenholz’s career was the development of a PEGylated liposomal doxorubicin formulation, known as Doxil^®^, which completely transformed care for cancer patients worldwide. Professor Barenholz’s research focuses on the biochemistry of lipids and membranes in addition to the biophysics laws underlying the fluidity of cellular membranes. Another focus area is the development of liposomes and lipid-based nanocarriers to overcome the shortcomings of current therapeutics by improving drug delivery. Professor Barenholz has authored more than 400 papers and has an h-index of 94; he is also the inventor of over 55 patents and an awardee of many prestigious national and international awards in the biomedical field. Professor Barenholz is highly regarded by his peers and students. One of the examples of his continuous contributions to the education of the next generation of scientists is the “Barenholz Prize”, which supports Israeli PhD students in applied sciences and encourages their professional growth and innovation. Professor Barenholz has founded and is currently leading the steering committee of the Hebrew University School of Business Administration BioMed-MBA program, through which he organized an online platform that enables the Israeli BioMed ecosystem.

This Special Issue comprises a collection of ten research and review articles prepared by international leaders in the fields of biomedical nanotechnology and drug delivery. The research articles describe several innovative technologies that span from the formulation of new nanomedicines and imaging agents to the in vitro assessment of their immunological properties.

The work presented by Professor Kim’s team from the Korea Institute of Science and Technology introduces new nanoparticles for combinational photochemotherapy of pancreatic cancer [1]. The formulation was based on light-activated monomethyl auristatin E prodrug linked to a photosensitizer (Ce6) through a caspase-3-specific cleavable peptide. Under irradiation with visible light, Ce6-generated reactive oxygen species induced the overexpression of caspase-3 in cancer cells, which, in turn, released the drug. The resulting formulations were extensively characterized, and in vivo data confirmed significant delays in tumor progression.

Professor Széchenyi, from the University of Pécs, and colleagues have developed a nanotechnology-based platform with which to treat onychomycosis [2]. The tested antifungal nanoformulations were based on silica-nanoparticle-stabilized Pickering emulsions, which were designed for the site-specific delivery of tioconazole and Melaleuca alternifolia essential oil. Microbiological in vitro experiments with relevant pathogens confirmed the significant antifungal effect and indicated promise for the topical treatment of onuchomycosis.

In order to overcome some of the commonly accepted hurdles associated with the clinical use of NIR dyes, the group of Professor Low from Purdue University has synthesized and tested a series of novel PEGylated UreterGlow derivatives [3]. The team identified promising bioimaging candidates with prolonged kidney retention times and unique emission profiles, not overlapping with other commonly used NIR probes.

Professors Yang and Kopeček, with colleagues from the University of Utah, have expanded the recently introduced notion of drug-free macromolecular therapeutics, or DFMT [4]. In their approach, the antibodies conjugated to short oligonucleotides could bind specific cell surface receptors, characteristic to diseased cells, and then become crosslinked via human serum albumin decorated with complementary oligonucleotides, which in turn induces apoptosis. The work published in this Special Issue demonstrated a scenario where DFMT was designed to crosslink CD38 receptors on lymphoma and multiple myeloma cells.

The immunology team of the Nanotechnology Characterization Laboratory at the Frederick National Laboratory for Cancer Research and Dr. Pang of the U.S. Food and Drug administration have evaluated the suitability of several in vitro assays that use peripheral blood mononuclear cells (PBMCs) as model systems to detect the innate immune responses induced by ten common immune-modulating impurities, as well as by a peptide drug product [5]. Based on the results of this comprehensive study, the sets of signature cytokines have been identified for further use in multiplex assays. In addition, the authors have demonstrated that the logistics of blood storage and handling must be taken into consideration and further evaluated, since they may influence the measured immunostimulatory responses.

Lastly, a collaborative effort between the Nanotechnology Characterization Laboratory at the Frederick National Laboratory for Cancer Research and Professor Afonin from the University of North Carolina at Charlotte have demonstrated how compositional variations in commercially available lipid-like carriers influence the immunostimulatory properties of nucleic acid nanoparticles that have different architectural characteristics [6].

The reviews cover several important topics that deal with the immunotoxicity of nanomedicines, drug formulations, and biomedical applications of nucleic-acid-based nanomaterials.

Dr. Stern and colleagues of the Nanotechnology Characterization Laboratory at the Frederick National Laboratory for Cancer Research provide a comprehensive review on nanomedicine reformulations of chloroquine and hydroxychloroquine to improve their therapeutic performance and broaden clinical applications [7]. Several reformulation nanomedicine approaches, ranging from liposomes to metal nanoparticles, have been discussed, as have the gaps in the current understanding of new nanoformulations; future perspectives and recommendations that may help to overcome the current limitations have been suggested.

Professor Chakrabarti and his team from the University of North Carolina at Charlotte discussed the ways how functional biological interactions can be studied using RNA nanotechnology [8], and Professor Afonin’s group from the same university elaborated on the immunorecognition of nucleic-acid-based nanoparticles designed for therapeutic applications [9].

Finally, the Nanotechnology Characterization Laboratory at the Frederick National Laboratory for Cancer Research reviewed the cellular and molecular mechanisms of inflammation caused by innate immunity-modulating impurities, with an emphasis on the safety and efficacy of pharmaceutical products [10].
